# The Core Stem Genes *SOX2*, *POU5F1/OCT4*, and *NANOG* Are Expressed in Human Parathyroid Tumors and Modulated by *MEN1*, *YAP1*, and β-catenin Pathways Activation

**DOI:** 10.3390/biomedicines9060637

**Published:** 2021-06-02

**Authors:** Chiara Verdelli, Annamaria Morotti, Giulia Stefania Tavanti, Rosamaria Silipigni, Silvana Guerneri, Stefano Ferrero, Leonardo Vicentini, Valentina Vaira, Sabrina Corbetta

**Affiliations:** 1 Laboratory of Experimental Endocrinology, IRCCS Istituto Ortopedico Galeazzi, 20161 Milan, Italy; chiara.verdelli@grupposandonato.it (C.V.); giulia.tavanti@unimi.it (G.S.T.); 2 Department of Pathophysiology and Organ Transplantation, University of Milan, 20122 Milan, Italy; annamaria.morotti@policlinico.mi.it (A.M.); valentina.vaira@unimi.it (V.V.); 3 Division of Pathology, Fondazione IRCCS Cà Granda Ospedale Maggiore Policlinico, 20122 Milan, Italy; stefano.ferrero@unimi.it; 4 Department of Biomedical, Surgical and Dental Sciences, University of Milan, 20122 Milan, Italy; 5 Medical Genetics Laboratory, Fondazione IRCCS Cà Granda, Ospedale Maggiore Policlinico, 20122 Milan, Italy; rosamariasilipigni@gmail.com (R.S.); silvana.guerneri@policlinico.mi.it (S.G.); 6 Endocrine Surgery, IRCCS Istituto Auxologico, 20122 Milan, Italy; viceleo@hotmail.com; 7 Endocrinology and Diabetology Service, IRCCS Istituto Ortopedico Galeazzi, 20161 Milan, Italy

**Keywords:** parathyroid tumors, *SOX2*, *NANOG*, *POU5F1/OCT4*, *MEN1*, *YAP1*, Wnt/β-catenin

## Abstract

Tumors of the parathyroid glands are the second most common endocrine neoplasia. Epigenetic studies revealed an embryonic signature involved in parathyroid tumorigenesis. Here, we investigated the expression of the stem core genes *SOX2, POU5F1/OCT4,* and *NANOG*. Rare cells within normal parathyroid glands expressed *POU5F1/OCT4* and *NANOG*, while *SOX2* was undetectable. Nuclear *SOX2* expression was detectable in 18% of parathyroid adenomas (PAds, *n* = 34) involving 5–30% of cells, while *OCT4* and *NANOG* were expressed at the nuclear level in a more consistent subset of PAds involving 15–40% of cells. Most parathyroid carcinomas expressed the core stem genes. *SOX2*-expressing cells co-expressed parathormone (PTH). In PAds-derived primary cultures, silencing of the tumor suppressor gene *MEN1* induced the expression of *SOX2*, likely through a *MEN1/HAR1B/SOX2* axis, while calcium-sensing receptor activation increased *SOX2* mRNA levels through *YAP1* activation. In addition, inducing nuclear β-catenin accumulation in PAds-derived primary cultures by short-term incubation with lithium chloride (LiCl), *SOX2* and *POU5F1/OCT4* expression levels increased, while *NANOG* transcripts were reduced, and LiCl long-term incubation induced an opposite pattern of gene expression. In conclusion, detection of the core stem genes in parathyroid tumors supports their embryogenic signature, which is modulated by crucial genes involved in parathyroid tumorigenesis.

## 1. Introduction

Tumors of the parathyroid glands are the second most frequent endocrine tumors and are often associated with parathormone (PTH) hypersecretion, determining primary hyperparathyroidism (PHPT). Parathyroid tumors are mostly benign and are considered a unique condition distinct from the more aggressive histology of the parathyroid atypical adenoma and parathyroid carcinoma. Several genes have been implicated in parathyroid tumorigenesis, most of them playing a tumor suppressor role. Accumulating evidence suggests the involvement of embryonic transcription factors in parathyroid tumors development. The precise expression of these transcription factors determines differentiation and migration of the parathyroid glands during embryogenesis [1]. Parathyroid embryonic transcription factors are also expressed in parathyroid cells during adult life. Notably, their expression is aberrant in parathyroid tumors: HOX genes are deregulated in parathyroid benign tumors [2], activating variants of the glial cell missing 2 (GCM2) gene have been identified in familial and sporadic cases of PHPT [3,4,5], and the T-box transcription factor 1 (TBX1) gene is downregulated in parathyroid tumors [6,7]. Moreover, an embryonic epigenetic signature has been detected in parathyroid tumors [8]: the microRNA cluster C19MC, which is highly expressed in embryonic stem cells, is aberrantly expressed [9].

Expression of the embryonic transcription factors is often associated with the expression of the early stem core genes *SOX2*, *POU5F1/OCT4,* and *NANOG*. These core genes are known as the core pluripotent transcription factors and are involved in several cell and developmental processes, including maintenance of embryonic stem cells [10]. These transcription factors bind to the enhancer regions of their target genes, modulating the expression levels of differentiation and self-renewal-related genes. Downregulation of the expression of the core stem genes induces differentiation of the embryonic stem cells. Normal adult cells do not express the core stem genes, while their expression is reactivated in a variety of human cancer cells [11]. In human cancers, the expression of the core stem genes identifies cancer stem cells, which are a unique population in the tumor, comprising 2–5% of the tumor bulk. Cancer stem cells share several features with embryonic stem cells. In particular, *NANOG* overexpression confers stemness, unlimited self-renewal, metastasis, invasiveness, angiogenesis, and drug-resistance by activating WNT pathway, *OCT4*, *SOX2*, and other genes [12]. The expression of the core stem genes has been reported in a variety of human cancers [11] and in benign neoplasia [13,14,15]. Of note, by NanoString nCounter assay exploring the expression of 740 genes involved in the four major processes of tumor progression (angiogenesis, extracellular matrix, epithelial–mesenchymal transition, and metastasis), *SOX2* gene expression has been recently detected in parathyroid cancers [7]. In addition, the core stem genes may be epigenetically modulated. For example, transcriptional activation of the C19MC cistron, which is overexpressed in a subset of parathyroid tumors [9], induces the expression of *OCT4* and accelerates cellular reprogramming [16].

Based on this evidence, the present study aimed to investigate the expression of the core stem genes in human parathyroid adenomas. Moreover, we hypothesized that the known tumor suppressor menin, encoded by the *MEN1* gene, whose expression is lost in multiple endocrine neoplasia (*MEN1*)-related parathyroid tumors [17], may be involved in the modulation of the core stem genes. Similarly, we investigated the potential role of the Hippo pathway-related YAP1 co-factor, whose nuclear expression has been recently found to be downregulated in parathyroid cancers [18], and the role of the WNT/β-catenin signaling. *YAP1* [19,20,21] and active β-catenin [22] have been reported to modulate the expression of the core stem cell genes.

Here, the core stem genes profile was investigated in normal and tumor parathyroid tissues by immunohistochemistry and immunofluorescence, confirming the deregulated embryonic signature of the parathyroid neoplasia. Moreover, modulation of the core stem genes has been analyzed, providing evidence of the involvement of the tumor suppressor genes *MEN1* and *YAP1* and of WNT/β-catenin signaling.

## 2. Materials and Methods

### 2.1. Tissue Samples

Twenty-four PAds from patients (19 females, 5 males; age 64.1 ± 2.25 years) with sporadic PHPT were collected and analyzed by RealTime PCR experiments. A further 20 PAds samples, with clinical and biochemical features similar to those of the PAds analyzed as whole tissue, were enzymatically dispersed; cells were cultured and used for gene silencing experiments, stimulation experiments, and immunofluorescence. Additionally, formalin-fixed paraffin-embedded (FFPE) sections were obtained from 5 normal parathyroid glands involuntary removed from normocalcemic patients surgically treated for thyroid diseases, 34 PAds for *SOX2* analysis, 4 PAds for *OCT4* analysis, 11 PAds for *NANOG* analysis, 5 parathyroid atypical adenomas (PAts), 3 parathyroid carcinomas (PCas; one metastatic cancer) for *SOX2* and *OCT4* analysis, and 8 PCas (2 metastatic cancers) for *NANOG* analysis were analyzed by immunohistochemistry (IHC). This study was approved by the Institutional Ethical Committee (Ospedale San Raffaele Ethical Committee, protocol no. GPRC6A PARA, approved on 07 March 2019; CE40/2019), and patients’ informed consent was obtained from all participants.

### 2.2. Immunohistochemistry

All archival tissue samples were cut and stained with hematoxylin and eosin for morphological assessment before immunohistochemistry (IHC). Then, tissue sections were incubated with primary antibody to *SOX2* (Clone D6D9XP^®^ Rabbit mAb, 1:100; Cell Signaling Technology, EuroClone, Milan, Italy), *OCT4* (Clone C30A3, 1:100; Cell Signaling Technology, EuroClone, Milan, Italy), *NANOG* (Clone D73G4, 1:100; Cell Signaling Technology, EuroClone, Milan, Italy), and *PTH* (BGN/1F8, sc-80924, Santa Cruz Biotechnology, DBA, Segrate, Italy). IHC was performed using Benchmark Ultra Roche Ventana Immunostainer (Roche Group, Tucson, AZ, USA) as previously described [23] and diaminobenzidine (DAB) was the chromogen. All slides were evaluated by an experienced pathologist (S.F.) and immunoreactivity was calculated as the percentage of positive cells out of the total number of parathyroid cells.

### 2.3. RNA Isolation and Real-Time Quantitative Reverse Transcription (qRT-PCR)

Total RNA was isolated from frozen tissues and PAds-derived primary cells using Trizol reagent (Invitrogen, ThermoFisher Scientific, Monza, Italy) following the manufacturer’s instructions. RNA was quantified by spectrophotometry at 260 nm (NANODROP ND-1000 Uv/Vis, Thermo Fisher Scientific, Whaltham, MA, USA) and DNA contamination was removed by DNase I (Life Technologies, ThermoFisher Scientific, Monza, Italy) treatment. Reverse transcription was performed using the iScript cDNA Synthesis Kit (Bio-Rad, Hercules, CA, USA) with a starting amount of 300 ng of digested RNA.

Real-Time PCR was conducted on a StepOne Plus System (ThermoFisher Scientific, Monza, Italy) using the following Taqman gene expression assays: *SOX2* (Hs01053049_s1), *OCT4/POU5F1* (Hs00999632_g1), *NANOG* (Hs02387400_g1), *AXIN2* (Hs00610344_m1), *DKK1* (Hs00183740_m1), *ZEB1* (Hs00232783_m1), *MEN1* (Hs00365720_m1), *HAR1B* (Hs03299152_m1), and *YAP1* (Hs00371735_m1) following the manufacturer’s protocol. Gene expression was quantified using a comparative Ct method and *HMBS* and *B2M* were used as housekeeping genes (Hs00609297_m1 and Hs99999907_m1, respectively) as previously described [24].

### 2.4. Primary Parathyroid Adenoma Cell Isolation and Culture

Samples from 8 PAds were cut into fragments less than 1 mm^3^, washed with PBS, and partially digested with Collagenase type I (Worthington, Lakewood, NJ, USA) 2 mg/mL for 90 min. Tissue digestion was filtered with a cell strainer (100 μm Nylon, BD Falcon, BioScientifica, Rignano Flaminio (RM), Italy) to obtain a single cell suspension. Cells were routinely grown at 37 °C in a humidified atmosphere of 5% CO_2_/air in Dulbecco’s Modified Eagle’s Medium (DMEM) supplemented with 10% FBS, 2 mM glutamine, and 100 U/mL penicillin-streptomycin.

### 2.5. DNA Extraction and Array Comparative Genomic Hybridization (aCGH) Analysis

Genomic DNA from 17 PAds was isolated using Trizol reagent (Invitrogen). The array-CGH analysis was performed using 60-mer oligonucleotide probe technology (SurePrint G3 Human CGH 8 × 60 K, Agilent Technologies, Santa Clara, CA, USA), according to the manufacturer instructions. The Feature Extraction and Cytogenomics 3.0.4.1, with the ADM-2 algorithm (Agilent Technologies), was used for data analysis. To improve results accuracy, the Diploid Peak Centralization algorithm was also applied. To determine aberrations, we set as the threshold a minimum of five consecutive probes/regions and a minimum absolute average log ratio (MAALR) of ± 0.25. To identify lower levels of mosaicism, a second analysis was run with a MAALR of ± 0.15. Only copy number variants not already reported in the public database of genomic variants (http://projects.tcga.ca/variation/ (accessed on 10 April 2021) were listed. The GRch37/hg19 of the Human Genome Reference (March 2009) consortium was used as the reference genome. Data were partially published previously [25].

### 2.6. Treatment of PAds-Derived Primary Cell Preparations with Lithium Chloride

PAds-derived single cells were treated with increasing concentrations of lithium chloride (LiCl, 10–20 mM) (Sigma-Aldrich, St. Louis, MO, USA), a known inhibitor of GSK3β kinase. After 8 and 72 h of treatment, cells were harvested and used for RNA analysis.

### 2.7. Cell Transfection and RNA Interference

For transient RNA interference experiments, PAds-derived primary cell cultures were seeded in 6-well plates at 1.5 × 10^5^ cells/well density. Cells were transiently transfected using Lipofectamine 3000 (Invitrogen-Thermo Fisher Scientific) in Opti-MEM (Gibco, Thermo Fisher Scientific), with *MEN1*-directed siRNA (EHU067451, Mission EsiRNA; Sigma Aldrich) or negative control siRNA (SIC001, Mission siRNA Universal negative control; Sigma-Aldrich) for 5 h, in accordance with the manufacturer’s instruction. *HAR1B* silencing was obtained using Dharmafect (T-2001-01; Dharmacon) as the transfection reagent and *HAR1B*-directed siRNA (SASI_Hs02_00378868; Sigma-Aldrich), while *YAP1* silencing was performed using Dharmafect (T-2001-01; Dharmacon) as the transfection agent and *YAP1*-directed siRNA (L-012200-00-005; ON-TARGET Plus siRNA SmartPool Dharmacon) or control siRNA (D-001810-10-05, ON-TARGET Non-Targeting Plus). siRNAs from Sigma Aldrich and Dharmacon are a mixture of different pre-designed siRNAs, all targeting the same mRNA sequence, minimizing the off-target effects. After 48 h, transfected cells were used for further experiments and analyzed by qRT-PCR.

### 2.8. Statistical Analysis

Data are presented as mean ± standard error of the mean (SEM). Non-normally distributed variables (failure of the Kolmogorov–Smirnov test) were log2 transformed. Correlations between two variables were tested with parametric or non-parametric tests as specified. Groups’ comparison was performed with one-way ANOVA adjusted for multiple comparisons. The *p* values less than 0.05 were considered statistically significant. All statistical analysis was performed using GraphPad Prism software v6.0 (GraphPad Inc., San Diego, CA, USA).

## 3. Results

### 3.1. The Core Stem Cell Genes SOX2, POU5F1/OCT4, and NANOG Are Expressed in Parathyroid Tumors

**Figure 1:**
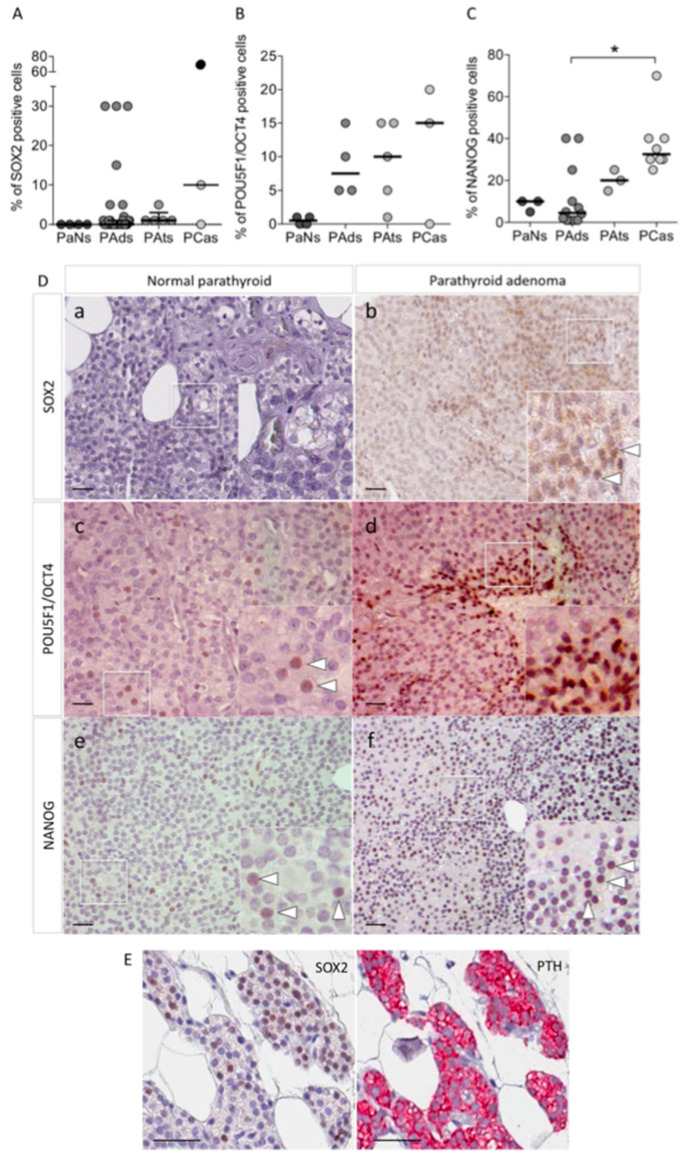
Expression of core stem genes in human parathyroid tissues. (**A**) Percentage of cells expressing *SOX2* detected by immunohistochemistry (IHC) in parathyroid tissues; black circle represents a metastatic PCa. PaNs, normal parathyroid glands; PAds, parathyroid adenomas; PAts, parathyroid atypical adenomas; PCas, parathyroid carcinomas. (**B**) Percentage of cells expressing *POU5F1/OCT4* detected by IHC in parathyroid tissues. (**C**) Percentage of cells expressing *NANOG* detected by IHC in parathyroid tissues; * *p* = 0.005 vs. PAds by Kruskall–Wallis test adjusted for multiple comparison. (**D**) Normal parathyroid cells did not show specific nuclear immunostaining for *SOX2* (**a**); PAds FFPE sections showed a subset of cells with weakly positive nuclear staining for *SOX2* (**b**). Nuclear staining for *POU5F1/OCT4* was detected in rare cells in normal parathyroid glands (**c**) (white heads of arrow), while *POU5F1/OCT4*-expressing cells were detected in cell clusters in PAds (**d**). Nuclear staining for *NANOG* was detected in rare cells in normal parathyroid glands (**e**) (white heads of arrow), and in a few PAds cells (**f**). All representative sections were acquired at 40× magnification and scale bars represent 200 nm. Inserts show specific nuclear staining. (**E**) Immunostaining of contiguous sections showing *SOX2*-expressing cells (**left**, brown) and the corresponding PTH staining (**right**, red); scale bars represent 200 nm.

The IHC analysis for *SOX2* failed to detect cells with specific nuclear staining in normal parathyroid glands (Figure 1A,D panel a and Supplementary Figure S1). This finding is in line with the Human Protein Atlas reports of IHC negative for *SOX2* in human normal parathyroid glands (www.proteinatlas.org (accessed on 10 April 2021)) and with the recent report by Condello et al. [7]. Similarly, most of PAds samples (*n* = 34) were negative for *SOX2* immunostaining (Figure 1A). Indeed, *SOX2*-expressing cells at a nuclear level were identified in 6 (18%) PAds, where the nuclear staining was detected in a proportion of cells variable from 5 to 30% of endocrine parathyroid cells (Figure 1A,D panel b) though the staining showed weak intensity. Similarly, *SOX2* nuclear expression was detected in few cells in 1 out 5 PAts, while one metastatic PCa expressed *SOX2* in 70% of cells (Figure 1A, black circle). Nuclei of the endothelial cells and fibroblasts in PAds were invariably negative for *SOX2*. In PAds, *SOX2*-expressing cells co-expressed *PTH* (Figure 1E).

At variance with *SOX2*, specific immunostaining for *POU5F1*/*OCT4* showed rare nuclear *OCT4*-expressing cells in normal parathyroid glands (Figure 1B,D panel c), while in the FFPE sections of PAds (*n* = 4), IHC identified 5–20% of cells with positive nuclear *OCT4* staining (Figure 1B,D panel d). *OCT4* was also detectable at nuclear levels in most PAts and PCas. Staining of FFPE sections with specific anti-*NANOG* antibodies identified rare scattered cells expressing *NANOG* protein at nuclear levels in normal parathyroid glands (Figure 1C,D panel e). In PAds, IHC detected positive *NANOG* nuclear staining in a proportion of cells ranging 1–40% (Figure 1C,D panel f), while all PAts and PCas samples showed a consistent proportion of cells, ranging from 20 to 40%, expressing *NANOG* at the nuclear level.

Considering PAds with available clinical data (*n* = 16), we found that PAds expressing the core genes transcripts were associated with circulating ionized calcium levels lower than those associated with undetectable transcripts (1.50 ± 0.02 vs. 1.61 ± 0.03 mmol/L; *p* = 0.039 by a Student’s *t*-test). No differences were detected in circulating PTH levels, age at diagnosis, or PAds weight between the two PAds groups (Table 1). Ionized calcium is the most reliable biochemical parameters reflecting parathyroid cell function.

### 3.2. Modulation of Core Stem Genes Expression in Sporadic Parathyroid Tumors

#### 3.2.1. Role of *MEN1*

**Figure 2:**
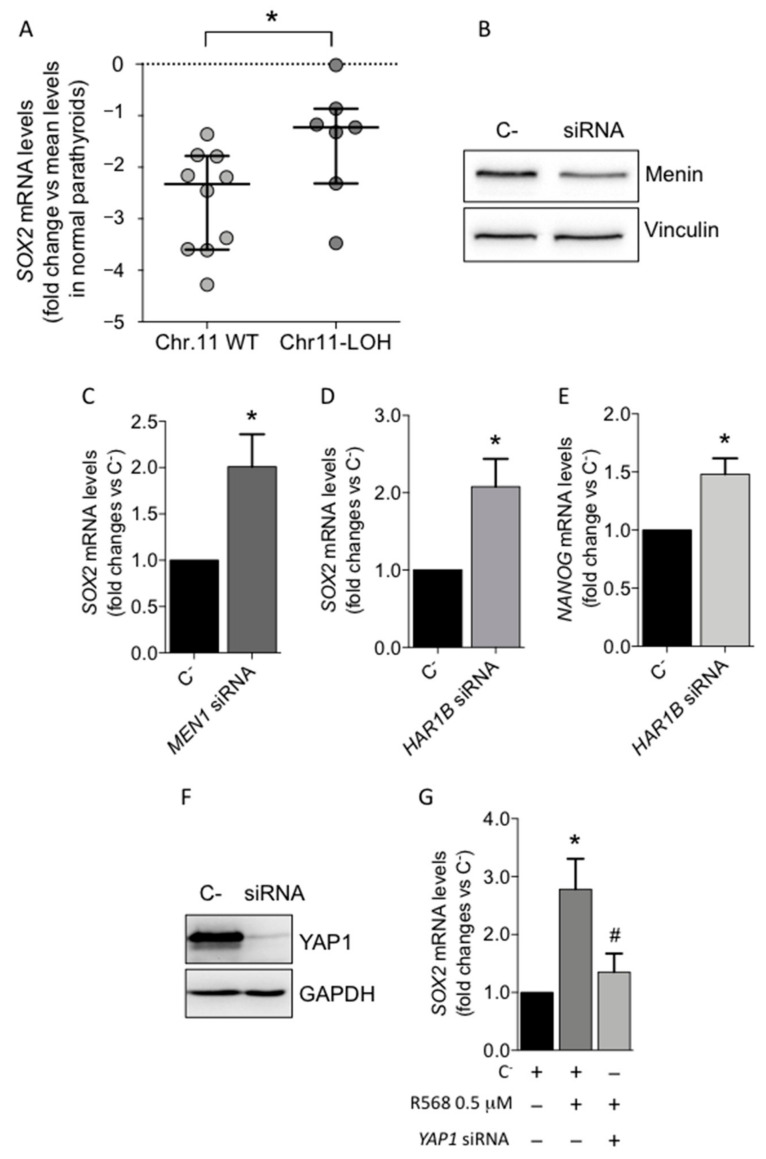
Effect of *MEN1* gene silencing and the CASR/YAP1 activated pathway on the expression of the core stem genes in PAds-derived cells. (**A**) *SOX2* gene expression levels according to PAds genetic background. Data are presented as log2 fold change with respect of the mean gene expression levels in normal parathyroid glands; bars represent mean and SEM; Chr 11-WT, PAds harboring both copies of chromosome 11; Chr 11-LOH, PAds harboring one copy of the chromosome 11; * *p* = 0.025. (**B**) *MEN1* gene silencing by smart pool siRNAs induced significant reduction of menin protein expression; (**C**) *SOX2* mRNA levels after *MEN1* silencing in PAds-derived cells; * *p* = 0.041 by one-way ANOVA adjusted for multiple comparison; data presented as mean ± SEM of four independent experiments. (**D**) Effect of *HAR1B* silencing on *SOX2* mRNA expression levels in PAds-derived cells (*n* = 3); (**E**) *HAR1B* silencing induced significant increases in *NANOG* mRNA levels. (**F**) *YAP1* gene silencing by smart pool siRNAs induced significant reduction of the YAP1 protein expression; (**G**) Effect of CASR activation on *SOX2* expression after 0.5 μM R568 treatment, with or without *YAP1* transient silencing in PAds-derived cells (*n* = 3). C-, PAds-derived cells treated with negative control siRNA; *MEN1* siRNA, PAds-derived cells treated with specific siRNA against *MEN1*; *HAR1B* siRNA, PAds-derived cells treated with specific siRNA against *HAR1B*; *YAP1* siRNA, PAds-derived cells treated with specific siRNA against *YAP1*; * *p* = 0.036; # *p* = 0.050.

We then analyzed the aCGH profiles in a set of 17 PAds and we correlated them with *SOX2* mRNA levels. Seven PAds harbored a monosomy of the chromosome 11 (Chr.11-LOH), detectable in 35–85% of cells. PAds harboring Chr.11-LOH expressed higher mRNA levels of *SOX2* compared with that of the PAds harboring biallelic Chr.11 (*p* = 0.025; Figure 2A). Considering that Chr.11-LOH occurs in about 40% of parathyroid adenomas and *MEN1* expression is variably conserved in parathyroid tumors, we chose to silence *MEN1* gene expression in order to evaluate the effect of its complete loss (Figure 2B). Transient silencing of the *MEN1* gene in PAds-derived cells (*n* = 4; Figure 2B) increased *SOX2* mRNA levels (Figure 2C), while it did not affect expression of both *POU5F1/OCT4* and *NANOG* (data not shown).

Moreover, we previously demonstrated that *MEN1* silencing increased the expression of a set of long non-coding RNAs (lncRNAs) in PAds [25]. We focused the attention on the lncRNA *HAR1B*, whose expression has been found to be upregulated in PAds harboring the Chr.11-LOH and has been upregulated by *MEN1*-silencing [25]. We tested the hypothesis that *HAR1B* mediates or contributes with menin to modulate the *SOX2* expression in parathyroid adenomas. Interestingly, *HAR1B* silencing induced an increase in *SOX2* (Figure 2D) and *NANOG* transcripts (Figure 2E), while *POU5F1/OCT4* expression was unaffected in PAds-derived cell preparations (*n* = 3).

#### 3.2.2. Role of CASR-Stimulated *YAP1* Signaling

The transcriptional co-activator *YAP1* is involved in the modulation of core stem genes [19,20,21]. A recent report showed that *YAP1* accumulation at the nuclear level is induced by calcium-sensing receptor (CASR) activation in PAds-derived cells [18]. In PAds-derived cells (*n* = 3), R568-mediated CASR activation increased *SOX2* mRNA levels, while the stimulatory effect was abolished by *YAP1* silencing (Figure 2F,G).

#### 3.2.3. Role of Canonical WNT/β-Catenin Signaling Pathway

**Figure 3:**
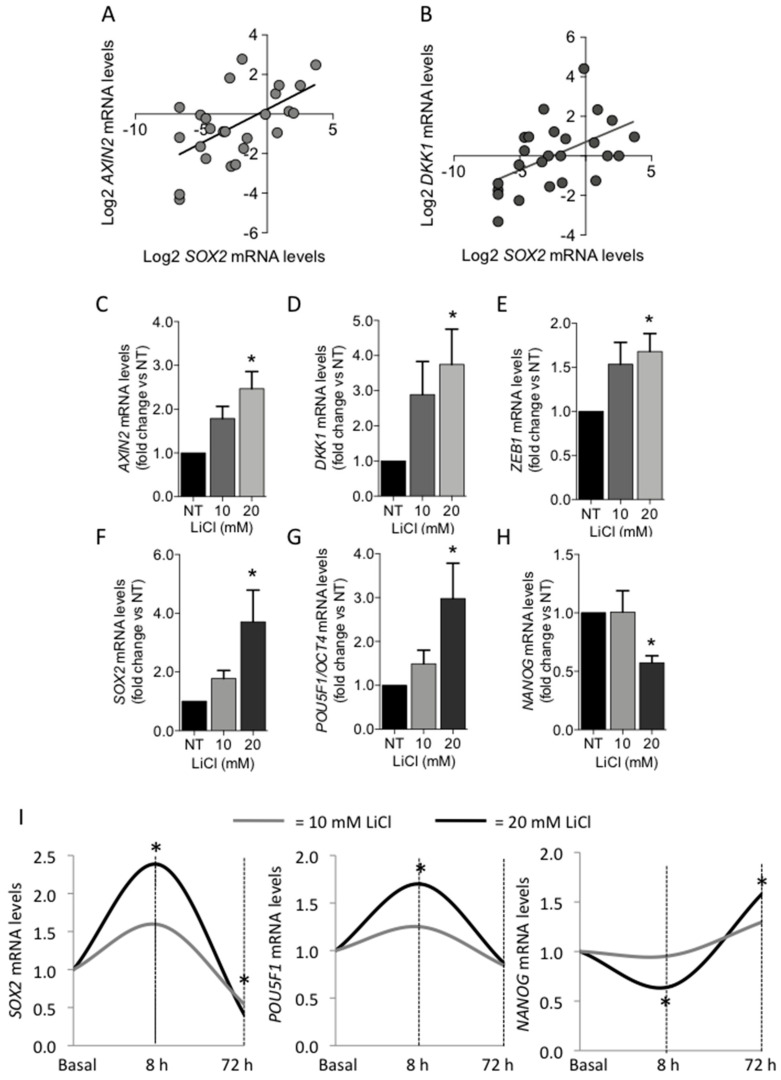
Effect of WNT/β-catenin activation on core stem genes expression in PAds-derived cells. (**A**) Positive correlation between *SOX2* and *AXIN2* mRNA levels. (**B**) Positive correlation between *SOX2* and *DKK1* mRNA levels; data are log2 transformed. Effects of LiCl treatment (10–20 mM) for 8 h in PAds-derived cells on *AXIN2* (**C**), *DKK1* (**D**), *ZEB1* (**E**), *SOX2* (**F**), *POU5F1/OCT4* (**G**), and *NANOG* (**H**) mRNA levels. (**I**) Effects of LiCl treatment (light grey lines, 10 mM; black lines, 20 mM) in PAds-derived cells on *SOX2*, *POU5F1/OCT4*, and *NANOG* mRNA levels after 8 and 72 h; * *p* < 0.05 by one-way ANOVA adjusted for multiple comparisons.

The canonical WNT/β-catenin pathway is intimately connected with pluripotency during embryogenesis [22]. Therefore, we investigated the effect of WNT/β-catenin activation on the expression of the core stem cell genes in PAds-derived cells. Positive correlations between *SOX2* and *AXIN2* mRNA levels (r = 0.586; *p* = 0.002) (Figure 3A) and between *SOX2* and *DKK1* mRNA levels (r = 0.519; *p* = 0.008) (Figure 3B) were detected.

Stimulation of PAds-derived cell cultures by increasing concentrations of lithium chloride (LiCl) (10–20 mM) for 8 h induced accumulation of active β-catenin at the nuclear level [6]. Here, we demonstrated that WNT pathway activation by LiCl for 8 h increased the β-catenin target genes *AXIN2* (Figure 3C; * *p* = 0.001 by one-way ANOVA adjusted for multiple comparison), *DKK1* (Figure 3D; * *p* = 0.068), and *ZEB1* (Figure 3E; * *p* = 0.057) in PAds-derived cells (*n* = 6). Moreover, LiCl (10–20 mM) induced increases in *SOX2* (*p* = 0.046; Figure 3F) and *POU5F1/OCT4* transcripts (Figure 3G; * *p* = 0.040), while *NANOG* mRNA expression levels were significantly reduced (Figure 3H; * *p* = 0.026). In contrast, by prolonging the treatment of PAds-derived cells (*n* = 3) with 10–20 mM LiCl for 72 h, *POU5F1/OCT4* mRNA levels returned to basal levels, while *SOX2* expression levels were downregulated and *NANOG* transcripts were significantly increased after 20 mM LiCl stimulation (Figure 3I).

## 4. Discussion

The present study provides evidence supporting the aberrant embryonic gene signature of parathyroid tumors. Besides deregulation of the transcription factors TBX1 [6] and GCM2 [5], which are involved in parathyroid embryonic differentiation, and of microRNAs belonging to the early embryonic pluripotent C19MC microRNA cluster [9], parathyroid tumors harbor cells expressing the core stem genes *SOX2*, *POU5F1/OCT4,* and *NANOG*. Recently, *SOX2* has been identified as significantly deregulated in parathyroid carcinomas [7]. In the present study, we focused our attention on the expression of *SOX2* and investigation was extended to the associated core stem genes *POU5F1*/*OCT4* and *NANOG*.

*SOX2* is a sex-determining region of a Y-related transcription factor, which belongs to a family of DNA-binding proteins [26]. *POU5F1*/*OCT4* is an octamer transcription factor, which binds to an eight-base pair DNA sequence and consists a Pit/Oct/Unc family of homeodomain proteins [27]. *SOX2* shares a common DNA-binding site with *POU5F1*/*OCT4* and has synergistic effects in regulation of their target genes by formation of a heterodimer. *NANOG* is a homeobox-containing transcription factor, and it is one of the target genes regulated by the heterodimer *SOX2*/*OCT4*. *NANOG* protein is expressed only in undifferentiated cells [12]. Notably, an interconnected *SOX2*/*OCT4*/*NANOG* regulatory circuit maintains embryonic stem cells self-renewal and pluripotency [28].

Cells expressing *SOX2* could not be detected in normal parathyroid glands from normocalcemic subjects, but normal glands showed rare cells expressing *OCT4* and *NANOG*. This observation resembles the few cells expressing core stem genes detected in normal oral epithelial cells or in cells of oral premalignant lesions [29]. Cells expressing the core stem genes were detected in a subset of parathyroid adenomas and in most parathyroid carcinomas. In line with data reported by Condello et al. [7], a metastatic parathyroid carcinoma expressed *SOX2* in about 70% of cells. In PAds, *SOX2*-expressing cells co-expressed PTH, suggesting that re-expression of the early embryonic stem cell factor does not affect the endocrine properties of the parathyroid cells. Of note, parathyroid adenomas expressing transcripts of the three core stem genes were associated with lower circulating ionized calcium levels when compared with that of tumors with undetectable gene transcripts.

We further investigated regulation of stem core genes expression in sporadic PAds. To this aim, we tested the hypothesis that regulatory molecules critical in parathyroid tumorigenesis, namely menin, encoded by the *MEN1* gene [30], calcium sensing receptor (CASR) [31], *YAP1* [18], and WNT/β-catenin [6], may be involved in modulation of the stem core genes. Chromosome 11 loss of heterozygosity (LOH) can be detected in at least 40% of parathyroid tumors [32] and *MEN1*, which maps on chromosome 11, is an important oncosuppressor involved in parathyroid tumorigenesis [33]. In sporadic PAds, tumors with chromosome 11 LOH showed higher *SOX2* transcript levels compared to that of PAds harboring biallelic chromosome 11. In line with this observation, reduction of *MEN1* expression increased *SOX2* mRNA expression levels in primary parathyroid adenomas cultures. *MEN1* silencing is also associated with deregulation of lncRNAs, among which *HAR1B* is the most significantly upregulated lncRNA in PAds harboring chromosome 11 LOHs compared with that of PAds with biallelic chromosome 11 [25]. Interestingly, *HAR1B* silencing in PAds primary cell cultures increased *SOX2* and *NANOG* transcripts, suggesting the existence of a *MEN1-HAR1B-SOX2/NANOG* regulatory axis.

Recently, it has been reported that CASR activation induces nuclear yes-associated protein 1 (*YAP1*) accumulation and transcription of *YAP1* target genes in PAds-derived cells [18]. The transcription factor *YAP1*, a major effector of the tumor suppressive Hippo signaling pathway, is necessary to maintain pluripotency in embryonic stem cells. High *YAP1* expression is particularly prominent in cancer stem cells. In osteosarcoma cells, *YAP1* can regulate the expression of *SOX2* by binding to two distinct DNA binding sites upstream and downstream of the *SOX2* gene [34]. In non-small cell lung cancers, *YAP1* transcriptionally induces *SOX2* through a physical interaction with *OCT4* [35]. In PAds-derived primary cell cultures, CASR activation by the selective agonist R568 increased the expression of *SOX2*, while *POU5F1/OCT4* and *NANOG* were unaffected. Reduction of *YAP1* nuclear accumulation by silencing *YAP1* [18] abolished the stimulatory effect of CASR activation on *SOX2* expression levels, thus suggesting that it is mediated by *YAP1* signaling activation. Considering that both CASR and *YAP1* feature as tumor suppressors in parathyroid adenomas, the induction of *SOX2* expression may promote cell resting more than cell proliferation [36].

The WNT/β-catenin pathway plays a pivotal role in the maintenance of human embryonic stem cells pluripotency and pluripotency exit and in somatic cell reprogramming [22]. β-catenin binds *POU5F1*/*OCT4* in embryonic stem cells [22], it stimulates human *NANOG* promoter activity [37] and it regulates *SOX2* activity in breast cancer cells [38]. Moreover, the expression of the core stem genes *SOX2*, *POU5F1*/*OCT4*, and *NANOG* is upregulated after β-catenin nuclear accumulation in embryonic stem cells [22].

The role of β-catenin in parathyroid tumorigenesis is not clear. Parathyroid tumors did not harbor constitutively activated β-catenin pathways: Activating mutations of the *CTNNB1*/β-catenin gene are rare and nuclear β-catenin immunostaining has not been demonstrated so far [39,40,41,42]. However, deregulation of WNT/β-catenin signaling modulators has been detected in parathyroid tumors compared to normal glands: Promoter hypermethylation of inhibitors of the WNT signaling *APC* and *RASSF1* [43,44], *SFRP1* [45,46], and *GSK3β* [40] have been described in both benign and malignant parathyroid tumors, while hypermethylation of *CTNNB1*/β-catenin occurs in about one fourth of PAds [47]. The effect of such deregulation in parathyroid tumor cells is still far from being elucidated [40,48].

In PAds, *SOX2* transcripts positively correlated with the WNT/β-catenin pathway target genes *AXIN2* and *DKK1*. In particular, AXIN2 is a well-known target of the canonical WNT signaling, and its expression provides a reliable readout of cells responding to WNT [49,50]. Here, we showed that WNT/β-catenin activation by LiCl, demonstrated by *AXIN2*, *DKK1,* and *ZEB1* increases, modulated the expression of *SOX2*, *POU5F1/OCT4,* and *NANOG* in PAds-derived cells. Tuning of the WNT signaling activity is of particular relevance as short-term stimulation induced an increase in *SOX2* and *POU5F1/OCT4* expression levels and reduced *NANOG* expression levels, while long-term stimulation inhibited *SOX2* expression and increased *NANOG* expression levels.

Present data provide a new perspective in parathyroid tumorigenesis, though the study suffers from some limitations: (1) modulation of the core stem genes has been mainly investigated in terms of mRNA levels due to difficulties in detection of the low amounts of core stem gene proteins expressed by few parathyroid cells; (2) tumor parathyroid cells expressing the early embryonic stem cell genes have not been isolated and functionally characterized; (3) it has not been elucidated whether core stem genes act separately or synergistically in tumor parathyroid cells; (4) the effects of *MEN1*, *YAP1*, and *HAR1B* overexpression on the core stem cell genes in tumor parathyroid cells have not been investigated; (5) the functional role of the core stem genes in tumor parathyroid cells has not been investigated.

## 5. Conclusions

Parathyroid tumors display an embryonic signature, providing new opportunities for the development of target therapy. Crucial genes in parathyroid tumorigenesis such as *MEN1*, *CASR,* and *YAP1* are regulators of the embryonic signature, suggesting new roles in parathyroid pathophysiology.

## Figures and Tables

**Table 1:** Comparison of clinical and biochemical parameters between parathyroid adenomas co-expressing core stem genes and parathyroid adenomas with undetectable core stem genes. *SOX2*, *POU5F1/OCT4*, and *NANOG* transcripts were detected by qRT-PCR in total RNA from tumor samples; data are log2 transformed and presented as mean ± SEM. Comparisons were analyzed by Student’s *t*-test. PAds, parathyroid adenomas; *n*, number; Ca^2+^, plasma ionized calcium; Ca, serum total calcium; PTH, plasma parathormone.

Parameters	PAds Co-Expressing Core Stem Genes mRNAs	PAds with Undetectable Core Stem Genes mRNAs	*p*
n	6	10	
*SOX2* mRNA levels	5.964 ± 1.836	0.024 ± 0.023	0.0008
*POU5F1/OCT4* mRNA levels	2.842 ± 0.527	−0.2920 ± 0.227	<0.0001
*NANOG* mRNA levels	3.727 ± 0.453	0.065 ± 0.070	<0.0001
Age (years)	51.0 ± 6.1	59.0 ± 2.8	0.197
Sex (female/male)	6/0	7/3	0.250
Plasma Ca2+ (mmol/L)	1.51 ± 0.02	1.61 ± 0.03	0.039
Serum total Ca (mg/dl)	11.5 ± 0.1	11.6 ± 0.3	0.977
Serum PTH (pg/ml)	231.2 ± 44.8	228.5 ± 47.8	0.971
Tumor size (cm)	1.70 ± 0.15	2.24 ± 0.40	0.312

## Data Availability

Supplementary Materials can be found at http://doi.org/10.5281/zenodo.4683021 (accessed on 10 April 2021).

## Supplementary Materials

The following are available online at https://www.mdpi.com/article/10.3390/biomedicines9060637/s1. Figure S1. IHC for *SOX2*. Upper panels represent normal parathyroid glands from normocalcemic patients negatively stained for *SOX2*. In panels a–c, nuclear positive IHC for *SOX2* of sections from human non-small cell lung cancer were used as the positive control. PaN, normal parathyroid gland.
