# Supplementary material for: The Core Stem Genes SOX2, POU5F1/OCT4, and NANOG Are Expressed in Human Parathyroid Tumors and Modulated by MEN1, YAP1, and β-catenin Pathways Activation
Source: Biomedicines. 2021 Jun 2;9(6):637. doi: 10.3390/biomedicines9060637 (PMC8227846; doi:10.3390/biomedicines9060637)
Supplement: Supplementary file 1 [file biomedicines-09-00637-s001.zip › biomedicines-1198654-supplementary.pdf]

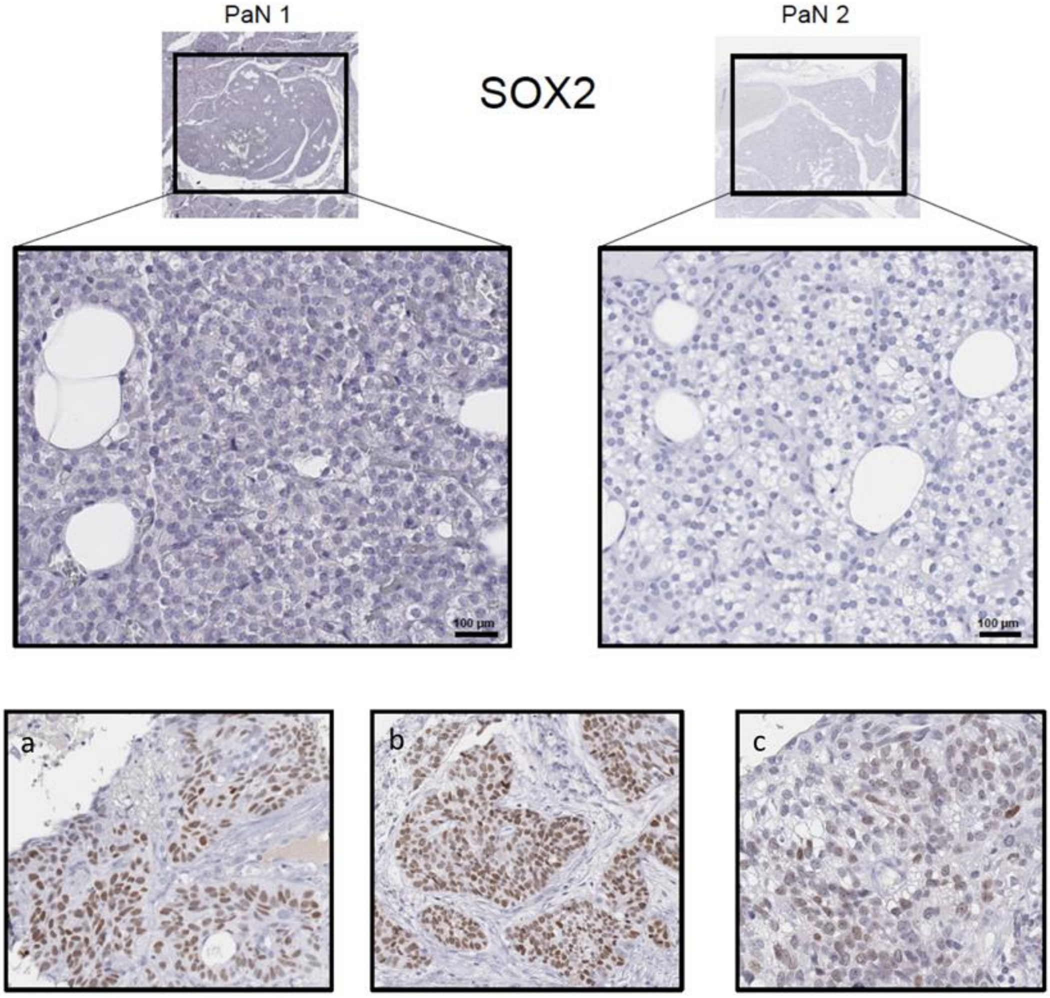

Supplementary Figure S1. IHC for SOX2. Upper panels represent normal parathyroid glands from normocalcemic patients negatively stained for SOX2. In panels a-c, nuclear positive IHC for SOX2 of sections from human non small cell lung cancer used as positive control. PaN, normal parathyroid gland.
